# The Children’s Urgent Reduction of Forearm Fractures in the Emergency Department (CURFFED) project: a national prospective trainee-led collaborative audit of practice

**DOI:** 10.1302/2633-1462.74.BJO-2025-0338.R1

**Published:** 2026-04-11

**Authors:** Lysander J. Gourbault, Robert Whitham, Andrew Womersley, Erin Jones, Alexander Christie, Luke Duggleby, William Guy Atherton, CURFFED Collaborative

**Affiliations:** 1 Paediatric Trauma and Orthopaedics, Bristol Royal Hospital for Children, Bristol, UK; 2 South West Surgery in Children Operational Delivery Network, Bristol, UK; 3 Royal United Hospital Bath NHS Trust, Bath, UK; 4 Gloucestershire Royal Hospital, Gloucester, UK; 1 Bristol Royal Hospital For Sick Children, Bristol, UK; 2 Broomfield, Essex, United Kingdom; 3 Chesterfield Royal Hospital, Chesterfield, United Kingdom; 4 Crosshouse Hospital, Kilmarnock, UK; 5 Derriford Hospital, Plymouth, UK; 6 Dorset County Hospital, Dorchester, UK; 7 East Surrey Hospital, Redhill, UK; 8 Frimley Park Hospital, Frimley, UK; 9 Great Western Hospital, Swindon, UK; 10 Huddersfield Royal Infirmary, Huddersfield, UK; 11 Ispwich Hospital, Ipswich, UK; 12 James Paget University Hospitals, Great Yarmouth, UK; 13 Kettering Hospital, Kettering, USA; 14 Kingston Hospital, Kettering, USA; 15 Lincoln County Hospital, Lincoln, UK; 16 Macclesfield District General Hospital, Macclesfield, UK; 17 Medway Maritime Hospital, Gillingham, UK; 18 Milton Keynes General Hospital, Milton Keynes, UK; 19 Musgrove Park Hospital, Taunton, UK; 20 New Cross Hospital, Wolverhampton, UK; 21 Norfolk & Norwich University Hospital, Norwich, UK; 22 North Devon District Hospital, Barnstaple, UK; 23 Poole Hospital, Poole, UK; 24 Prince Charles Hospital, Chermside, Australia; 25 Queen Alexandra Hospital Portsmouth, Portsmouth, UK; 26 Queen Elizabeth Hospital (Woolwich), London, UK; 27 Queen's Hospital Romford, Romford, UK; 28 Royal Albert Edward Infirmary, Wigan, UK; 29 Royal Alexandra Hospital for Sick Children, Brighton, UK; 30 Royal Bolton Hospital, Bolton, UK; 31 Royal Cornwall Hospital, Truro, UK; 32 Royal Devon & Exeter Hospital, Exeter, UK; 33 Royal Gwent Hospital (Newport) / Aneurin Bevan, Newport, UK; 34 Royal Hospital for Sick Children Glasgow, Glasgow, UK; 35 Royal Hospital for Sick Children in Edinburgh, Edinburgh, UK; 36 Royal Surrey Hospital, Guildford, UK; 37 Royal Sussex County Hospital Brighton, Brighton, UK; 38 Royal United Hospital, Bath, UK; 39 Russell's Hall Hospital, Dudley, UK; 40 Salisbury District Hospital, Salisbury, UK; 41 Sandwell District General Hospital, West Bromwich, UK; 42 Southampton University Hospitals NHS Trust, Southampton, UK; 43 St Richard's Hospital & Worthing Hospital, Chichester, UK; 44 St Richard's Hospital, Chichester, UK; 45 Stoke Mandeville, Aylesbury, UK; 46 Torbay Hospital, Torquay, UK; 47 Tunbridge Wells, Kent, UK; 48 University Hospital of Coventry & Warwickshire, Coventry, UK; 49 University Hospital of North Staffordshire, Stoke-on-Trent, UK; 50 Warwick Hospital, Warwick, UK; 51 Wexham Park Hospital, Slough, UK; 52 William Harvey Hospital Ashford, Ashford, UK; 53 Wishaw Hospital, Wishaw, UK; 54 Worthing, Worthing, UK; 55 Wythenshawe Hospital, Manchester, UK; 56 Yeovil District Hospital, Yeovil, UK; 57 Milton Keynes University Hospital, Milton Keynes, UK

**Keywords:** Forearm fractures, Paediatric, Trauma, Wrist fractures, BOAST, Forearm Fractures, wrist fractures, paediatric forearm fractures, forearms, analgesia, pain scores, distal radius fractures, British Orthopaedic Association, paediatric fractures

## Abstract

**Aims:**

This audit aimed to assess compliance with British Orthopaedic Association Standards for Trauma (BOAST) for paediatric forearm and wrist fractures across UK NHS hospitals and identify targets for improvement locally and nationally.

**Methods:**

This was a prospective, multicentre observational audit of BOAST standards for the Early Management of the Paediatric Forearm Fracture guideline. Consecutive patients aged under 16 years presenting with a forearm or distal radius fracture over a two-month period were included with follow-up to eight weeks post injury. Data were collected to assess each of the BOAST standards for practice. Percentage compliance with all standards was calculated for each hospital.

**Results:**

Data from 1,699 patients across 53 hospitals were included. The mean age was 9.7 years (SD 3.6), and 37% (n = 636) were female. Overall, 60% of fractures (n = 1,023) were metaphyseal distal radius fractures. A total of 577 patients (34%) underwent manipulation with the majority initially reduced in the Emergency Department (ED) (n = 423, 73%); 89 (21%) required subsequent theatre manipulation. The median time to first manipulation in the ED was two hours 43 minutes (IQR 1 hr 43 mins to 4 hrs 4 mins) and 18 hours 47 minutes (IQR 13 hrs 48 mins to 24 hrs 2 mins) when first manipulation was performed in theatre. Overall compliance with BOAST standards was 63%, with 20% of patients (n = 85) having pain scores documented, 51% (n = 217) having a complete neurovascular assessment, and 23% (n = 95) receiving analgesia and a patient information leaflet on discharge.

**Conclusion:**

This study highlights variability in managing paediatric fractures despite established standards. In line with recommendations, a high proportion of reductions are now being performed in EDs. Particular areas requiring improvement are the management of paediatric pain, documented assessment of neurovascular status, and the provision of patient information. We recommend that hospitals review their current practice and ensure that local protocols are in place to promote the provision of optimal care for this patient group, and to minimize the impact on operating theatre capacity.

Cite this article: *Bone Jt Open* 2026;7(4):531–539.

## Introduction

The forearm and wrist are the most common sites of fracture in children, accounting for approximately one-third of fractures.^1,2^ The annual incidence of paediatric forearm and wrist fractures varies from 56 to 227 per 100,000 person-years dependent on patient age, most commonly occurring between the ages of five and nine years.^3^

Paediatric patients’ capacity for bony remodelling allows for an acceptance of deformity greater than fractures in adults, especially in the upper limb.^4^ However, where forearm and wrist fractures demonstrate a degree of deformity or displacement that is perceived to exceed remodelling potential, early closed reduction and casting using appropriate analgesia or conscious sedation is often the treatment of choice.^5^

A British Orthopaedic Association (BOA) Standard for Trauma (BOAST) was published in 2021, aiming to standardize practice for angulated, but not off-ended, paediatric forearm fractures.^6^ The BOAST aims to improve care of patients with these injuries within Emergency Departments (EDs) by encouraging early manipulation, definitive casting, and avoidance of admission for a procedure under general anaesthesia. Nevertheless, the 2022 Getting It Right First Time (GIRFT) programme review into NHS paediatric orthopaedic services highlighted wide variation in the proportion of forearm and wrist fractures being managed outside of the ED environment.^7^

A potential 250 weeks of operating time a year could be saved by improving protocols for forearm and wrist fracture manipulation in EDs, allowing for more effective use of limited theatre resources.^7^ Previous studies have demonstrated the positive effect of implementing protocols for manipulating forearm and wrist fractures in EDs, but no studies have assessed implementation of the BOAST standard nationally.^8,9^

The Children’s Urgent Reduction of Forearm Fractures in the Emergency Department (CURFFED) audit aimed to assess compliance with BOAST standards for paediatric forearm and wrist fractures across UK NHS hospitals and identify areas of practice that can be targeted for improvement at local and national levels.

## Methods

This study was a prospective, multicentre observational audit of BOAST guidelines on the Early Management of the Paediatric Forearm Fracture.^6^ Consecutive patients aged under 16 years presenting with a forearm or distal radius fracture over a two-month period (between 1 March 2024 and 30 April 2024) were included with follow-up to eight weeks post injury. Adult patients (aged ≥ 16 years), and patients presenting to the ED more than 48 hours following the injury, buckle fractures, and off-ended fractures were excluded.

Data were collected to assess each of the BOAST standards for practice, and included presence of departmental policies regarding paediatric forearm and wrist fractures, documentation of neurovascular status, administration and assessment of pain relief, use of X-rays, and use of manipulation (Supplementary Material, Appendix 1). Percentage compliance was calculated for each hospital using Excel (Microsoft, USA).

Overall compliance to the BOAST standard was calculated out of a score of 10 and a percentage compliance was calculated. Each of the specific standards was allocated a score of one and, where partially met, a score of 0.5 was given (i.e. for present but incomplete documentation for neurovascular status and provision of analgesia and advice).

### Ethical approval

A Health Research Authority (HRA) decision tool was completed confirming that this study is not considered research and no formal ethical approval was required. Each local centre was required to register the audit with their hospital’s Clinical Audit department and seek approval from their Caldicott Guardian to share anonymized data with the audit leads. Data were anonymized and collated by the South West Surgery in Children Operational Delivery Network, then shared with the central team via NHS email with data encryption compliant with international data protection guidelines.

## Results

### Demographic data

**Fig. 1:**
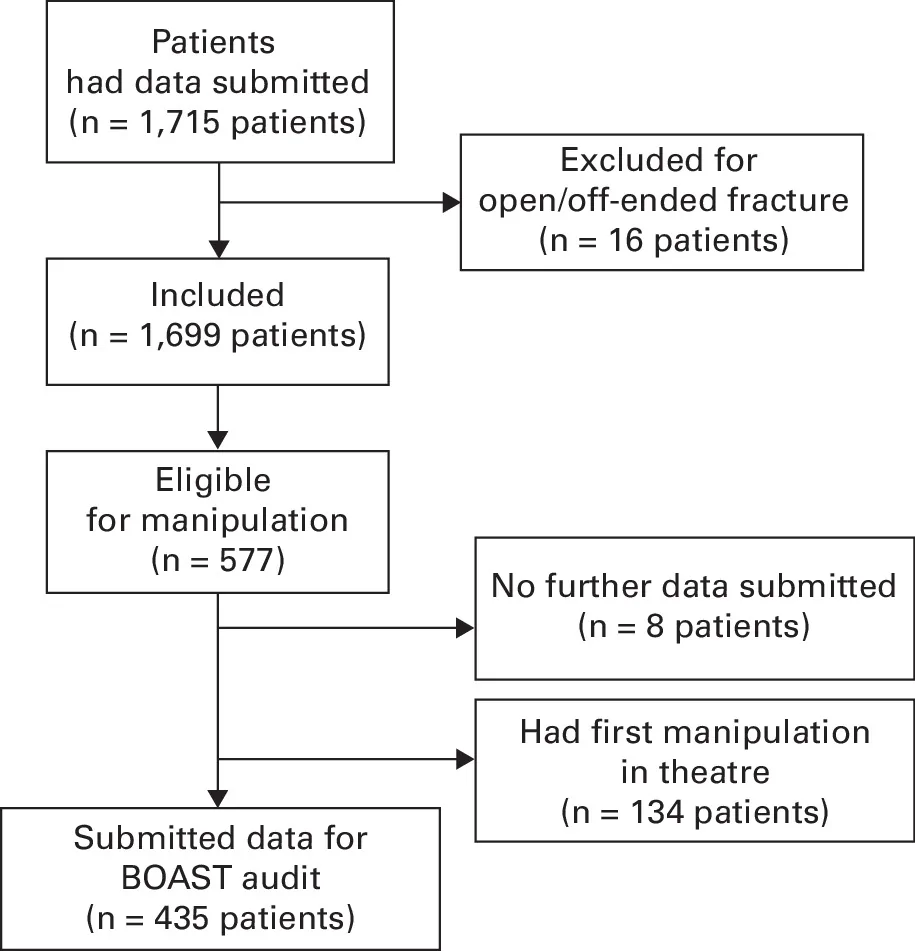
Flowchart illustrating data inclusion.

**Fig. 2:**
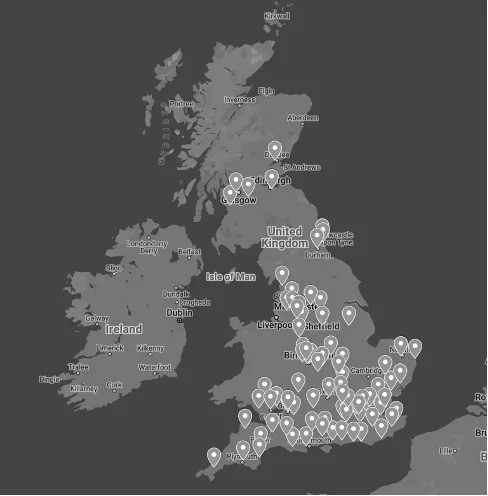
Map of the UK with data collection sites (grey markers).

**Fig. 3:**
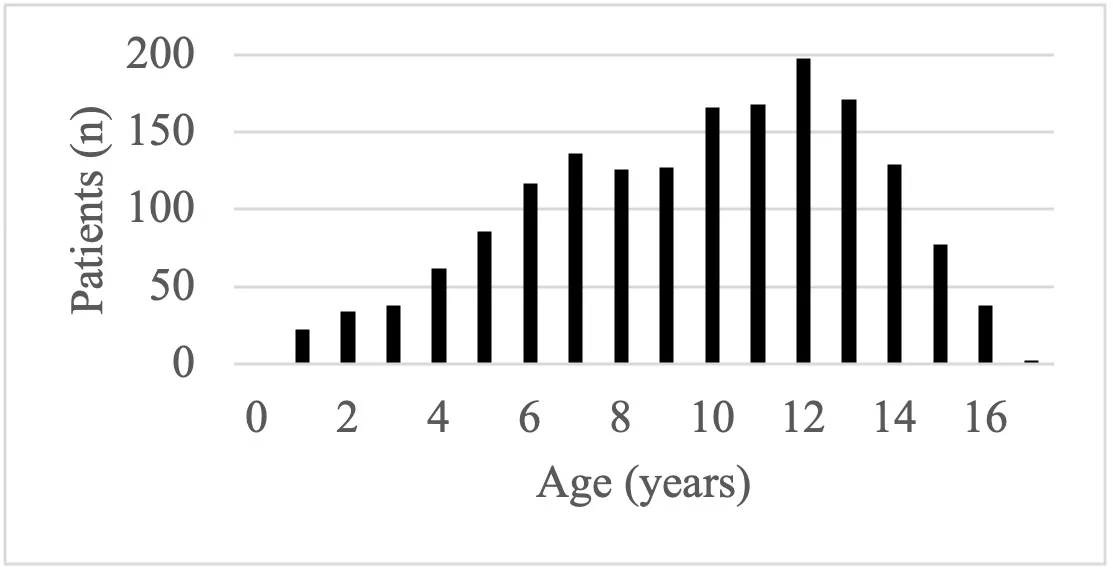
Histogram showing the age of patients included in the Children’s Urgent Reduction of Forearm Fractures in the Emergency Department audit.

Data from 1,715 patients were submitted from 53 sites across Great Britain, including eight of the 19 (42%) UK Major Trauma Centres (MTCs; Figure 1 and Figure 2). There were 16 patients who were excluded for having open and/or off-ended fractures, as per the inclusion criteria. Of the remaining 1,699 patients, 1,063 were male (63%) and 636 were female (37%) with a mean age of 9.7 years (SD 3.6) (Figure 3). The most common anatomical location of the fracture was metaphyseal (60.2%, n = 1,023; Table I).

**Table I.** Fracture diagnosis.

Fracture diagnosis	Patients, n (%)
Diaphyseal	393 (23)
Intra-articular	8 (0.5)
Metaphyseal	1,023 (60)
Salter Harris I & II	248 (15)
Other	26 (2)

Of the 1,699 patients, 577 (34%) were deemed eligible for manipulation in the ED. The majority of those eligible had at least one attempt at manipulation performed in the ED (n = 423, 73%). For an overview of manipulation location, please refer to Table II.

**Table II.** Initial manipulation location.

Location	Patients, n (%)
Emergency Department	335 (59)
Emergency Department followed by operating theatre	89 (16)
Operating theatre	134 (24)
Fracture clinic	8 (1)

**Fig. 4:**
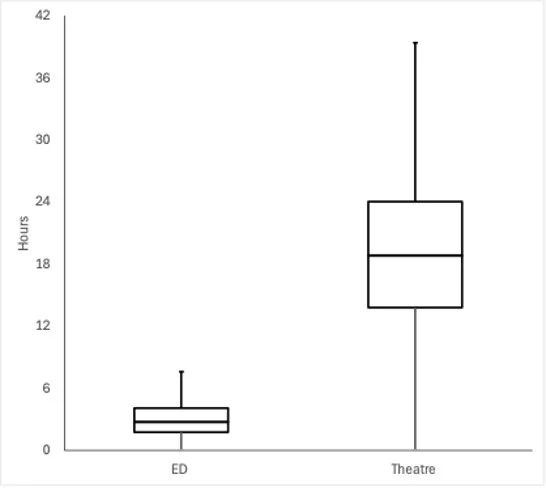
Box plot of time to first manipulation by location. ED, Emergency Department.

Median time to first manipulation in the ED was two hours 43 minutes (IQR 1 hr 43 mins to 4 hrs 3 mins) and if first manipulation was performed in theatres this was 18 hours 47 minutes (IQR 13 hrs 48 mins to 24 hrs 2 mins; Figure 4). For those patients manipulated in fracture clinic, the median time to manipulation was 9 days (IQR 1.7 to 10; Table III). The mean total number of departmental radiographs taken for patients manipulated in ED was 8.4 (SD 4.1), for ED and theatre 8.8 (SD 4.7), and theatre 7.1 (SD 3.9). The mean number of radiographs taken for patients manipulated in the fracture clinic was lower at 4.6 (SD 1.9), however there were only eight patients primarily manipulated in this location (Table IV).

**Table III.** Mean time to first manipulation by location.

Location (n)	Time (hours:minutes)
ED (423)	5:19
Theatre (134)	28:28
Fracture clinic (5)	150:07
Median (IQR)	3:21 (2:01 to 8:11)

**Table IV.** Mean number of radiographs.

Location (n)	Mean (SD)
Emergency Department (335)	8.4 (4.1)
Emergency Department and operating theatre (89)	8.8 (4.7)
Operating theatre (134)	7.1 (3.9)
Fracture clinic (5)	4.6 (1.9)
Mean, regardless of location (563)	8.1 (4.2)

### BOAST standard compliance

The first standard for practice within this BOAST states that all centres should have local guidelines for the management of paediatric forearm fractures; of the 53 sites that submitted data, nine provided local protocols and eight provided a copy of their hospital patient information leaflet. Documented pain scores were available for 20% of patients (83/417). Prescription of regular analgesia was documented for 87% of patients (n = 372 of 426). Parental or patient consent was documented for 38% (163/426). For radiological assessment of the fractures, pre-manipulation radiographs were obtained in 99% of cases (424/429) and post-reduction radiographs in 98% (419/427). A pre-manipulation neurovascular assessment was documented for 91% of patients (387/426). The assessment was complete (i.e. contained discrete documentation for individual nerves and pulse) in 50% of cases (214/426). A post-manipulation neurovascular assessment was documented for 61% of patients (257/423) and this was complete in 35% of cases (147/423). Provision of oral analgesia and/or advice was documented for 81% of patients (343/417). A review of the case by a consultant within 48 hours was documented for 78% of patients (328/423) and 59% were seen in a fracture clinic within seven days (238/405).

**Fig. 5:**
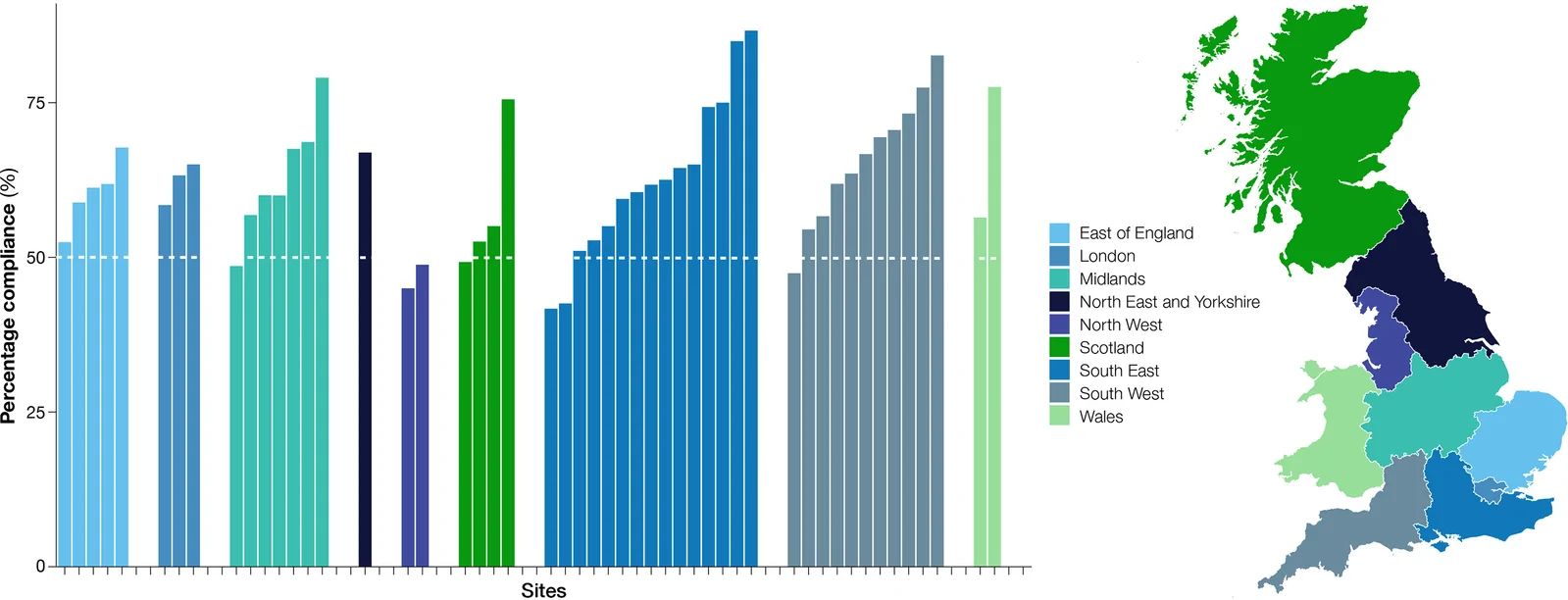
Bar chart of percentage BOAST compliance by hospital, and divided by region.

A total of 50 hospitals submitted sufficient data to calculate an overall percentage compliance to BOAST. The mean compliance was 63% (42.5% to 86.9%; Table V). Figure 5 demonstrates the ranges of compliance by hospital and region.

**Table V.** BOAST compliance.

Standard	Achieved, n (%)
Pain scores documented	83 (20)
Regular analgesia prescribed	372 (87)
**Pre-manipulation NV status documented**	
Yes - complete	214 (50)
Yes - incomplete	173 (41)
**Post-manipulation NV status documented**	
Yes - complete	147 (35)
Yes - incomplete	110 (26)
Consent process documented	163 (38)
Pre-manipulation radiographs	424 (99)
Post manipulation radiographs	419 (98)
**Discharged with analgesia and advice**	
Yes - complete	95 (23)
Yes - incomplete	243 (58)
Analgesia only	45 (11)
Advice only	198 (47)
Consultant review of case within 48 hrs	328 (78)
Clinic within 7 days	238 (59)

NV, neurovascular.

## Discussion

This was a national collaborative audit of BOAST standards for the early management of paediatric forearm fractures across 53 UK NHS hospitals. It demonstrated variation in practice across the country with an overall compliance of 63%. Of the 1,699 patients included, 577 (34%) underwent manipulation with the majority initially reduced in the ED (n = 424, 75%).

The main objective of the BOAST standards and GIRFT was to increase the number of reductions performed in the ED for the benefit of the patient.^6,7^ In this audit, performing the reduction in ED reduced the median delay to manipulation from 18 hours 47 minutes if performed in theatre to two hours 43 minutes in the ED. Early reduction reduces the distress experienced by families from a return visit and preparation for surgery.^3^ Additionally, the economic benefit is estimated to be £1.6 million per annum to the NHS.^7^ This target could be further achieved by using mini-C-arm fluoroscopy for ED reductions of paediatric forearms, which has been shown to reduce the need for further reduction or interventions in theatre.^10^

In 2022, 250 weeks of operating time across the country were estimated to be used for manipulations of paediatric forearm fractures; this is now estimated to be reduced to 100 weeks, since the introduction of the BOAST standard.^7,11^ Our study showed that 424 (75%) of initial manipulations are being performed in the ED, rising to 458 (81%) in hospitals with defined protocols, highlighting the progress achieved. There is still room for improvement. In 45 (32%) of cases the reason the manipulation was not done in the ED was due to resource limitations, lack of staff, or lack of space; in 44 (31%) of cases the reason to reduce in theatre was a clinical one. Sufficient resourcing of our EDs is therefore essential.

The decision not to reduce in the ED often relates to the lack of capacity to perform sedation and concern of clinicians that intranasal opioids do not provide sufficient analgesia to perform fracture reduction.^12^ However, with building evidence for their effectiveness and acceptability to both patients and their caregivers, the use of an intranasal opioid and Entonox for procedural analgesia may help reduce the reliance on operating theatres.^5,8,13^ This transition may be further helped by the use of methoxyflurane as an adjunct to intranasal opioids, with the recent MAGPIE trial showing it to be safe to use in the paediatric population, and to more significantly reduce patient pain scores compared to Entonox. The MAGPIE study may pave the way for UK licensing and more widespread adoption of this analgesic method.^14^

Forearm and wrist fractures are painful injuries which require appropriate analgesia. This audit has shown that although 372 (87%) of patients received analgesia, only 83 (20%) had their pain levels documented. This is inadequate, as children’s pain is frequently undertreated and a comprehensive pain assessment is essential.^15,16^ Repeated Royal College of Emergency Medicine (RCEM) audits of “Pain in Children” have highlighted this issue, showing delays to assessing levels of pain and providing timely analgesia.^17^ It is therefore vital that units put in place the recommendations from the RCEM which include the use of pain scores which staff have been trained in, and systems to ensure that pain is re-evaluated.^17^

Variation in clinical practice is widespread in healthcare and while it can be useful in the presence of clinical equipoise, when clear guidelines are set it can be unwarranted and detrimental to patients.^18,19^ This study found variation in follow-up timing and consultant review of manipulated forearm and wrist fractures: only 238 (59%) of patients had follow-up within one week, and 328 (78%) had a consultant review within 48 hours. Early consultant review is important to identify loss of reduction, especially where the potential for remodelling of the fracture is debated.^20^

Obtaining informed consent is a fundamental medical, legal, and ethical principle, and patients should be aware of the potential benefits and risks of the options available to them.^21^ This study has shown poor recording of formal consent (38% (163/426)), and complete assessment of neurovascular status (50% (214/426)) prior to forearm manipulation. Although it is likely that patients are consented verbally prior to manipulation, written consent is best practice and less open to scrutiny. Regarding the documentation of neurovascular status, our findings are similar to a national audit of UK ankle fracture management in which 59% of patients had complete documentation.^22^ However, our results show higher levels of documentation than a study assessing the perioperative neurovascular status of supracondylar humerus fractures (11%).^23^ Although only 0.7% of paediatric forearm fractures have an associated nerve injury, identification is essential so that appropriate treatment can be initiated.^24^ Failure to document appropriately not only puts patients at risk of harm, but is also a key cause of litigation.^25^ With poor documentation implicated in 40% of the £180 million paid out by NHS resolutions in 2017/2018 regarding orthopaedic surgery, it is imperative that clinicians prioritize comprehensive documentation.^25,26^ This could be improved by using standardized neurovascular assessment proformas.^23,27,28^

As per the GMC’s Good Medical Practice, it is essential that patients and their families receive information about their diagnosis in a comprehensible way, including details regarding diagnosis, prognosis, treatment options, and what to do should complications arise.^21^ Our study showed that only 47% of patients are provided with documented advice, and only eight centres were able to find a relevant patient information leaflet (PIL) to provide to patients. While verbal information is important, it has been shown that patients immediately forget 40% to 80% of this, and only half of the information they retain is correct.^29,30^ This can be improved by 50% when text information is also provided, although it is essential that it is written in a way that the general population can understand.^29-31^ We commend the BOA’s start on collating exemplary PILs, and hope that this work will be developed further.^32^

This study has several limitations. Despite its prospective nature, it relies on documentation which may have been inaccurate or absent, including patients followed up at different units to their index presentation. Not all patients may have been identified, especially those not requiring a manipulation, as these do not always attend fracture clinic follow-up. It is likely that more centres have protocols and PILs than 17% and 15% respectively, however there is clearly a lack of awareness as to their existence or where to find them which needs improving. As an audit, we were unable to collect any specific outcome data assessing the impact of compliance with BOAST guidance on patient outcome.

To our knowledge, this is the largest prospective audit of national practice regarding paediatric forearm and wrist fractures in the UK. By utilizing a national trainee-led collaborative model, we were able to collect data from across the country including 53 EDs, eight of which are MTCs (42% of paediatric MTCs in the UK). This allowed our data to be representative of current UK practice, and for areas of improvement to be identified including consent process, neurological assessment, and follow-up.

This national prospective collaborative audit of BOAST standards demonstrated variable compliance. However, a high proportion of reductions are now being performed in EDs in line with recommendations. Particular areas for improvement are the documentation of neurovascular status, provisions of PILs, and management of paediatric pain. We recommend that local departments continue to regularly review their current practice ensuring that local protocols are in place to minimize the impact on operating theatre capacity and promote the provision of optimal care for this patient group.

**Take home message**

- The CURFFED project was a prospective, multicentre observational audit of BOAST standards for the Early Management of the Paediatric Forearm Fracture guideline.

- This study demonstrated variability in managing paediatric forearm fractures despite established standards, and highlights key areas of improvement.

## Data Availability

The data that support the findings for this study are available to other researchers from the corresponding author upon reasonable request.
